# Tracking intratumoral heterogeneity in glioblastoma via regularized classification of single-cell RNA-Seq data

**DOI:** 10.1186/s12859-020-3390-4

**Published:** 2020-02-18

**Authors:** Marta B. Lopes, Susana Vinga

**Affiliations:** 1 Instituto de Telecomunicações, Instituto Superior Técnico, Universidade de Lisboa, Av. Rovisco Pais 1, Lisboa, 1049-001 Portugal; 2 INESC-ID, Instituto Superior Técnico, Universidade de Lisboa, Rua Alves Redol 9, Lisboa, 1000-029 Portugal

**Keywords:** Glioblastoma, Sparse logistic regression, Gene network, Twiner

## Abstract

**Background:**

Understanding cellular and molecular heterogeneity in glioblastoma (GBM), the most common and aggressive primary brain malignancy, is a crucial step towards the development of effective therapies. Besides the inter-patient variability, the presence of multiple cell populations within tumors calls for the need to develop modeling strategies able to extract the molecular signatures driving tumor evolution and treatment failure. With the advances in single-cell RNA Sequencing (scRNA-Seq), tumors can now be dissected at the cell level, unveiling information from their life history to their clinical implications.

**Results:**

We propose a classification setting based on GBM scRNA-Seq data, through sparse logistic regression, where different cell populations (neoplastic and normal cells) are taken as classes. The goal is to identify gene features discriminating between the classes, but also those shared by different neoplastic clones. The latter will be approached via the network-based twiner regularizer to identify gene signatures shared by neoplastic cells from the tumor core and infiltrating neoplastic cells originated from the tumor periphery, as putative disease biomarkers to target multiple neoplastic clones. Our analysis is supported by the literature through the identification of several known molecular players in GBM. Moreover, the relevance of the selected genes was confirmed by their significance in the survival outcomes in bulk GBM RNA-Seq data, as well as their association with several Gene Ontology (GO) biological process terms.

**Conclusions:**

We presented a methodology intended to identify genes discriminating between GBM clones, but also those playing a similar role in different GBM neoplastic clones (including migrating cells), therefore potential targets for therapy research. Our results contribute to a deeper understanding on the genetic features behind GBM, by disclosing novel therapeutic directions accounting for GBM heterogeneity.

## Background

Tumor heterogeneity is a major bottleneck in cancer diagnosis and therapy, playing a critical role in cancer invasion, metastasis and therapy resistance [1]. Glioblastoma (GBM), the most common primary brain malignancy in adults and one of the most aggressive cancers [2], is an archetypal example of a heterogeneous cancer, exhibiting extensive cellular and molecular heterogeneity, both within and between tumors [3, 4]. Current treatments combining surgery with radiotherapy and chemotherapy programs have shown to prolong survival, however, tumor recurrence usually occurs within two years [5]. Recurrence has been mainly attributed to the diffuse nature of GBM, with infiltrating neoplastic cells originating from the tumor core spreading quickly across long distances within the brain, rendering local therapies ineffective [5].

Transcriptome analysis has been extensively used to classify tumors into molecular subtypes and to establish signatures to predict the response to therapy and patient outcomes [6]. While bulk tumor sequencing is arguably powerful in classifying GBM subtypes [7], it becomes clearly ineffective when it comes to identify and characterize rare cell populations, e.g., infiltrating neoplastic cells in GBM patients. Gene expression by bulk cell populations dilutes the contribution of these rare cells to the overall gene expression pattern [8], thus representing a confounding factor in clinical diagnosis and therapeutic treatment of patients [9]. With the advances in next-generation sequencing and single-cell RNA sequencing (scRNA-Seq) it is now possible to get into the cell level and tackle intratumoral heterogeneity [3, 5, 10–13]. Not only cancer cells, but also non-cancerous cells that, together with the extracellular matrix form the tumor macroenvironment, can be fully investigated, as they are known to shape the progression of cancer and are deeply involved in the patient outcome [6].

Inter- and within-tumor heterogeneity in GBM has been previously described through scRNA-Seq analysis [3, 5]. In the study by Darmanis at al. (2017) [5], besides a large degree of heterogeneity between and within four different tumors, the analysis revealed a population of infiltrating neoplastic cells originating from the peripheral tissue whose transcriptional and genomic variant profiles resembled tumor core cells. Notably, infiltrating GBM cells were found to share a consistent gene signature across highly variable tumors. These findings open new directions for therapy research, targeting not only neoplastic cells in general, but also infiltrating populations of cells migrating away from the primary tumor, responsible for recurrence [5].

Alongside the remarkable advances in technology and biomarker discovery, there is a continuous demand for the development of statistical and machine learning methods able to translate the vast amounts of data retrieved by next-generation sequencing technologies into a clinically application format [14]. scRNA-Seq datasets comprise tens of thousands genes and irrelevant information that render ill-posed models. Sparsity-inducing models are a common strategy to cope with the high-dimensionality problem as in scRNA-Seq data. Standard sparsity is usually enforced through the *l*_1_ regularizer, i.e., the least absolute shrinkage and selection operator (LASSO) [15], which in the presence of strongly correlated variables may only select one out of the highly correlated set of variables. Since genes are organized in co-expression networks, selecting subnetworks of interrelated genes might be more appropriate when modeling RNA-Seq data. The elastic net (EN) regularizer [16], a combination of the *l*_1_ and the *l*_2_ norms, stands as a valuable alternative to the LASSO for highly correlated scenarios.

Aiming at the identification of disease gene signatures in GBM, regularizers can be used in the models loss function to select the relevant features in the discrimination between different GBM clones, providing hints on key drivers on tumor progression and therapy resistance. Regularizers can also be coupled with prior information on the underlying genes network, with the premise that network information yields more interpretable and reproducible models [17, 18]. In this context, the twiner regularizer has been recently proposed to extract common gene RNA-Seq signatures in cancers with similarities at the molecular level, by imposing a lower penalty on genes showing a similar correlation pattern in the genes correlation networks of the diseases under study. For instance, it is pertinent to evaluate whether known subnetworks present in two diseases are indeed selected as relevant in a classification scheme where the two diseases are a class against, e.g., a non-disease class. The result is a shared disease signature between diseases. The twiner regularizer showed promising results in the identification of a common gene signature in breast and prostate cancer [17], with associations to survival time distributions in both cancers.

Extending the scope of application of twiner to track tumor heterogeneity based on scRNA-Seq data seems particularly promising in biomarker selection in GBM. The possibility of identifying genes signatures shared by the different tumor clones, e.g., neoplastic cells from the tumor core and infiltrating neoplastic cells originated from the tumor periphery, could unravel putative disease biomarkers to target multiple neoplastic clones.

We propose a procedure based on a classification setting to discriminate between different cell groups in GBM tumors, including neoplastic and normal cells from the tumor core, and neoplastic cell from the tumor periphery. The results obtained are expected to fulfill a three-fold goal: i) disclose gene signatures in discriminating between neoplastic and normal cells; and ii) identify putative molecular drivers that provide infiltrating neoplastic cells with the capabilities for migrating through a non-tumor environment; iii) identify shared disease signatures between different neoplastic tumor clones irrespective of their tumor location.

The dataset obtained by Darmanis et al. (2017) [5] will be used in this study, consisting of scRNA-Seq data obtained from four GBM patients. Binary sparse logistic regression using the EN and the twiner penalties will be use for the designed classification scenarios. The gene selected shall be regarded as putative disease biomarkers in the resolution of GBM heterogeneity as well as in the design of multi-clone target therapies.

## Results

**Fig. 1:**
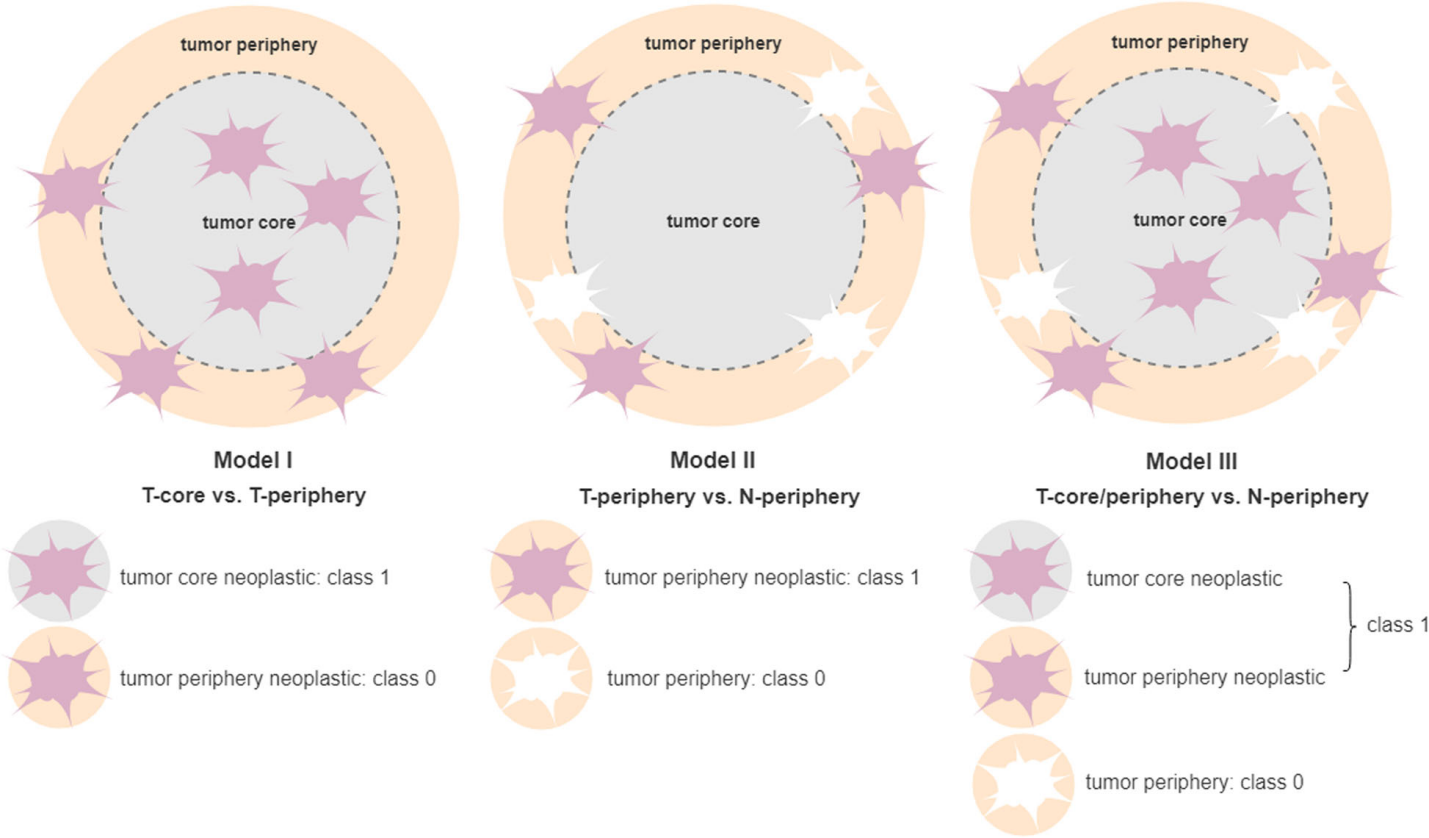
Schematic representation of the selection of the cell types as classes (1 and 0) to build the datasets to be used in the classification models: Model I (T-core vs. T-periphery), Model II (T-periphery vs. N-periphery), and Model III (T-core/periphery vs. N-periphery)

Three sparse classification models were built aiming at extracting gene signatures from scRNA-Seq GBM data (Fig. 1). The model results regarding the median number of variables selected and the accuracy measures obtained for the 1000 bootstrap samples can be found in Table 1. Overall, a high accuracy was obtained for the three models, with AUC values ≥0.94, a low number of misclassifications and a comparable median number of genes selected.

**Table 1:** Median accuracy results obtained from the application of Models I, II, and III to the 1000 bootstrap samples generated (T, tumor neoplastic astrocytes; N, normal astrocytes; EN, elastic net; NB, Naïve Bayes: MSE, mean squared error; AUC, area under the precision-recall curve; Miscl, misclassifications; Vars, nr. of variables selected)

Classes	Model	Vars	Miscl		MSE		AUC	
			Train	Test	Train	Test	Train	Test
I - T-core vs. T-periphery	EN	83	10	7	0.029	0.047	0.97	0.94
II - T-periphery vs. N-periphery	EN	85	3	4	0.020	0.037	0.99	0.96
III - T-core/periphery vs. N-periphery	EN	76	1	2	0.005	0.012	0.997	0.982
	Twiner	76	0	2	0.003	0.011	1	0.982
	NB_*EN*_	76	5	6	0.009	0.034	0.996	0.979
	NB_*twiner*_	76	4	5	0.008	0.028	0.996	0.981

**Fig. 2:**
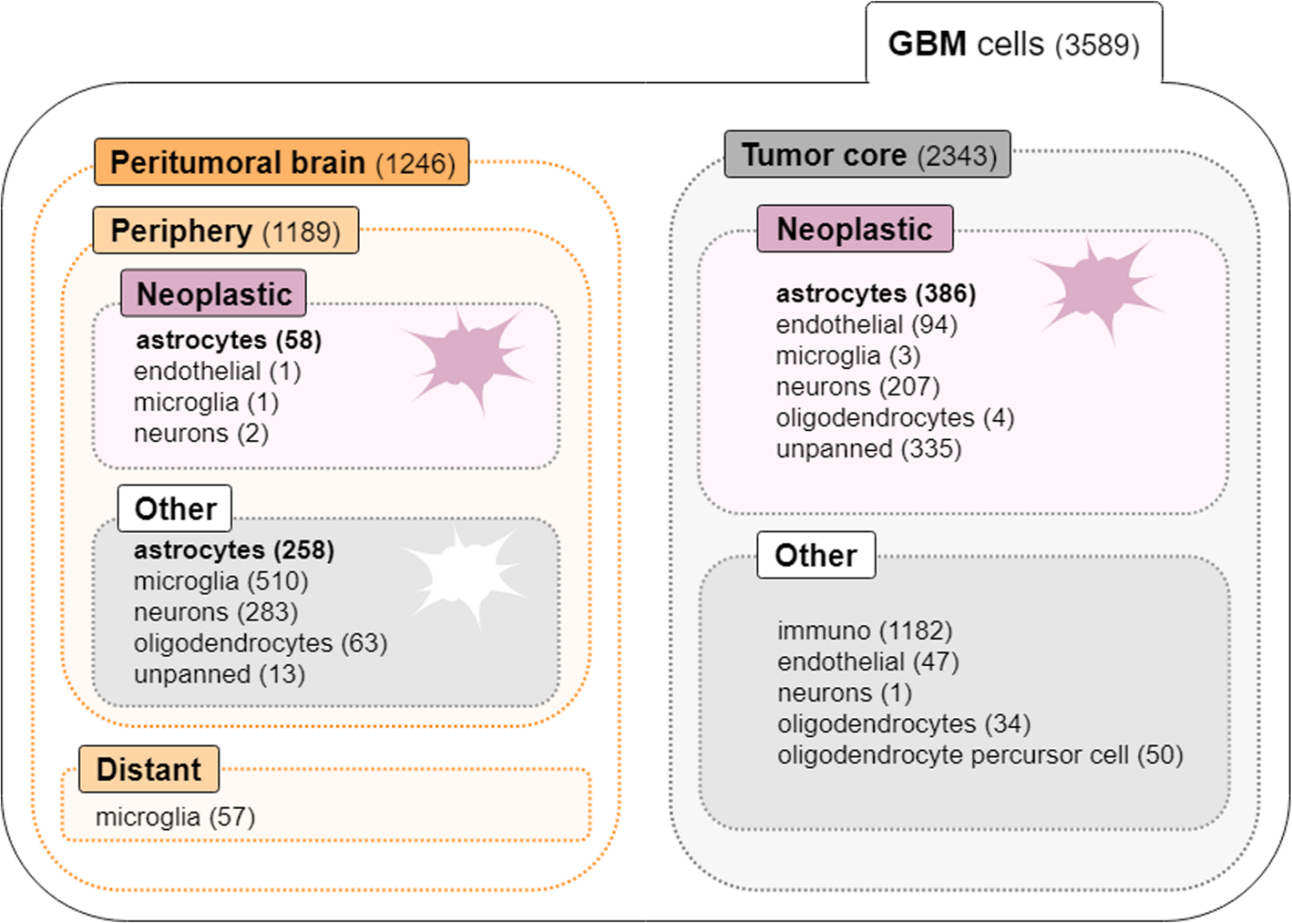
Data summary on the number of cells in each category regarding cell type and location

Model I was generated by sparse logistic regression based on the EN penalty to classify cells into neoplastic astrocytes from the periphery, i.e., infiltrating neoplastic cells, and the tumor core. The goal was to identify gene features that discriminate between the two cell populations, particularly those enabling tumor neoplastic cells to migrate from the tumor core to the peritumoral space. Model I presented a higher number of misclassifications compared to Models II and III, which besides the higher number of samples cells considered (*n*=444; Fig. 2) might be related to the increased difficulty in distinguishing between periphery neoplastic (infiltrating) astrocytes and tumor core neoplastic astrocytes, showing marked molecular similarities. A total median number of 83 genes were selected as relevant in the discrimination between the two classes, from which 15 were selected in more than 75% of the 1000 model runs (Table 2). From those, *ATP1A2* and *PRODH* were always selected. All genes were up-regulated in neoplastic periphery (infiltrating) astrocytes, except *PCSK1N* and *TMSB10*, which were down-regulated.

**Table 2:**
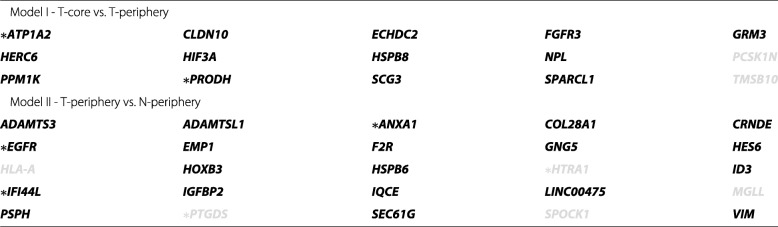
Genes selected in more than 75% of the 1000 runs by Models I and II (T, tumor neoplastic astrocytes; N, normal astrocytes); bold and gray coloured genes are up- and down-regulated, respectively, in neoplastic periphery astrocytes (T-periphery) against neoplastic tumor core astrocytes (Model I) and normal periphery astrocytes (Model II); genes marked with an asterisk are genes that were selected in the 1000 model runs

Model II was designed to disclosing cancer drivers that make astrocytes from the periphery distinguishable in neoplastic and normal cells. Similarly to Model I, it was built based on sparse logistic regression with the EN penalty. A median number of 85 genes were selected in across the bootstrap samples generated (Table 1). Twenty-five genes were selected in more that 75% of the 1000 models, from which 5 (*ANXA1*, *EGFR*, *HTRA1*, *IFI44L*, and *PTGDS*) were always selected (Table 2). The majority of the genes were up-regulated in neoplastic periphery (infiltrating) astrocytes, except *HLA-A*, *HTRA1*, *MGLL*, *PTGDS*, and *SPOCK1*, which were down-regulated.

**Fig. 3:**
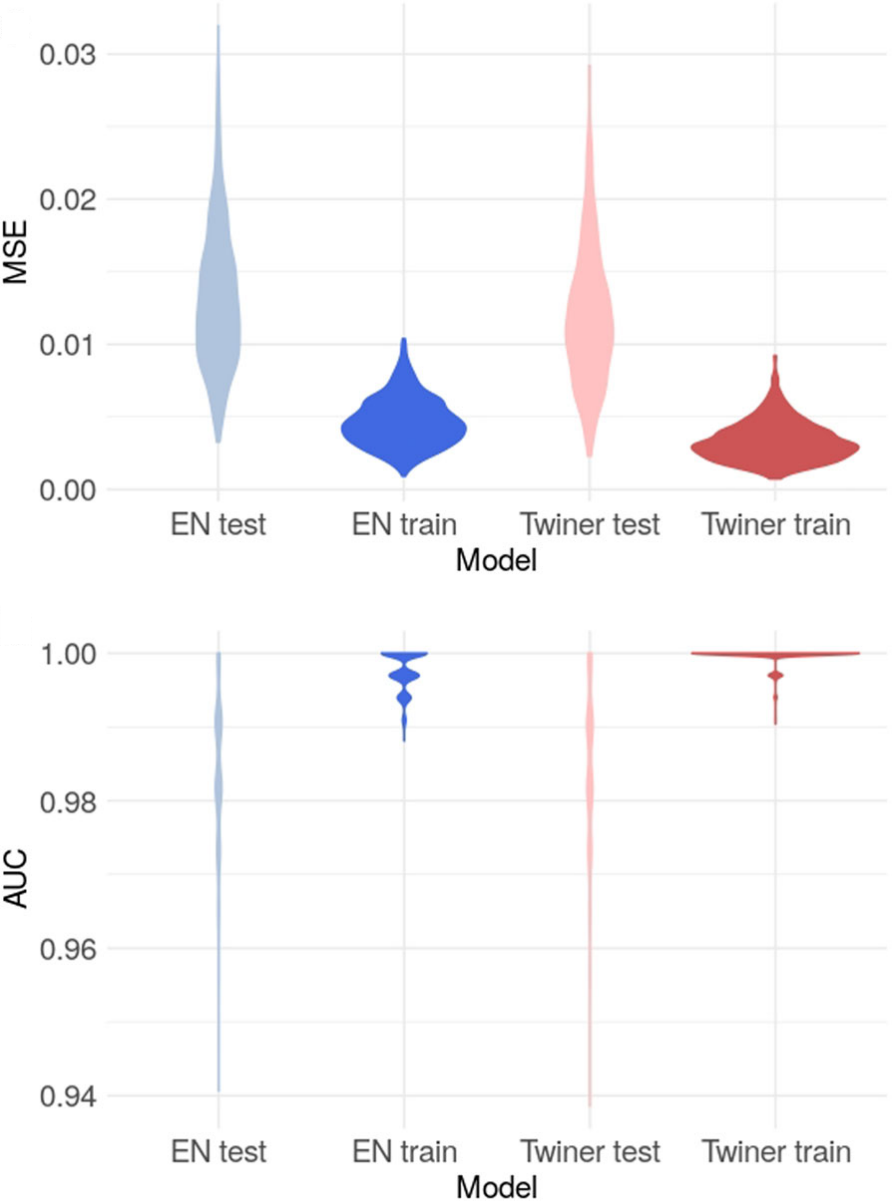
Accuracy measures obtained for the 1000 sparse logistic regression models generated via EN and the twiner regularization (MSE, mean squared error; AUC, area under the curve), for the train and test sets, considering the ‘T-core/periphery vs. N-periphery’ case study (Model III)

A different classification strategy was adopted for Model III to classify GBM astrocytes into neoplastic (tumor and periphery) and normal astrocytes, with the goal of identifying shared molecular signatures between neoplastic astrocytes from different tumor locations, putative biomarkers to target GBM heterogeneity. Regularization in the sparse logistic model was enforced via the EN and the twiner penalties, the later enabling the identification of the genes that are similarly correlated in neoplastic astrocytes from both the periphery and tumor core, and that play a role in the discrimination between neoplastic (tumor and periphery) and normal astrocytes. Sparse classification via twiner regularization yielded slightly better performance regarding the MSE and AUC over the 1000 model runs compared to EN (Table 1; Fig. 3), with a median increased performance in MSE of 29% in the training set and 11% in the test set. The same median number of variables was selected by the two modeling approaches, i.e., 76 variables.

For model comparison with a benchmark method, the set of variables selected by EN and twiner were used in the NB classifier. For these model scenarios, a slightly decreased accuracy was obtained for the NB classifier (Table 1).

**Fig. 4:**
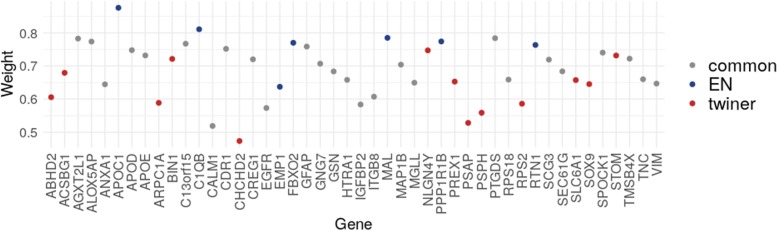
Weights of the variables (genes) selected by sparse logistic regression via EN and twiner regularization, considering the ‘T-core/periphery vs. N-periphery’ case study (Model III); the variables are colored differently whether they are selected exclusively by EN (blue) or twiner (red), or selected in common by the two methods (gray)

A total of 39 genes were selected by twiner in more that 75% of the runs, from which 26 genes were selected in common with EN (Fig. 4). Thirteen genes were exclusively selected by twiner, showing a comparatively lower weight regarding the genes selected by EN, thus confirming the ability of twiner to select genes with a similar role in the correlation networks of neoplastic cells from the periphery and tumor core. Regarding the genes included in the twiner signature, the following 8 genes were always selected: *APOD*, *CDR1*, *EGFR*, *HTRA1*, *IGFBP2*, *MGLL*, *PTGDS*, and *SEC61G*, some previously selected by Model II, also classifying GBM cells into neoplastic (from the tumor periphery) and normal astrocytes.

**Fig. 5:**
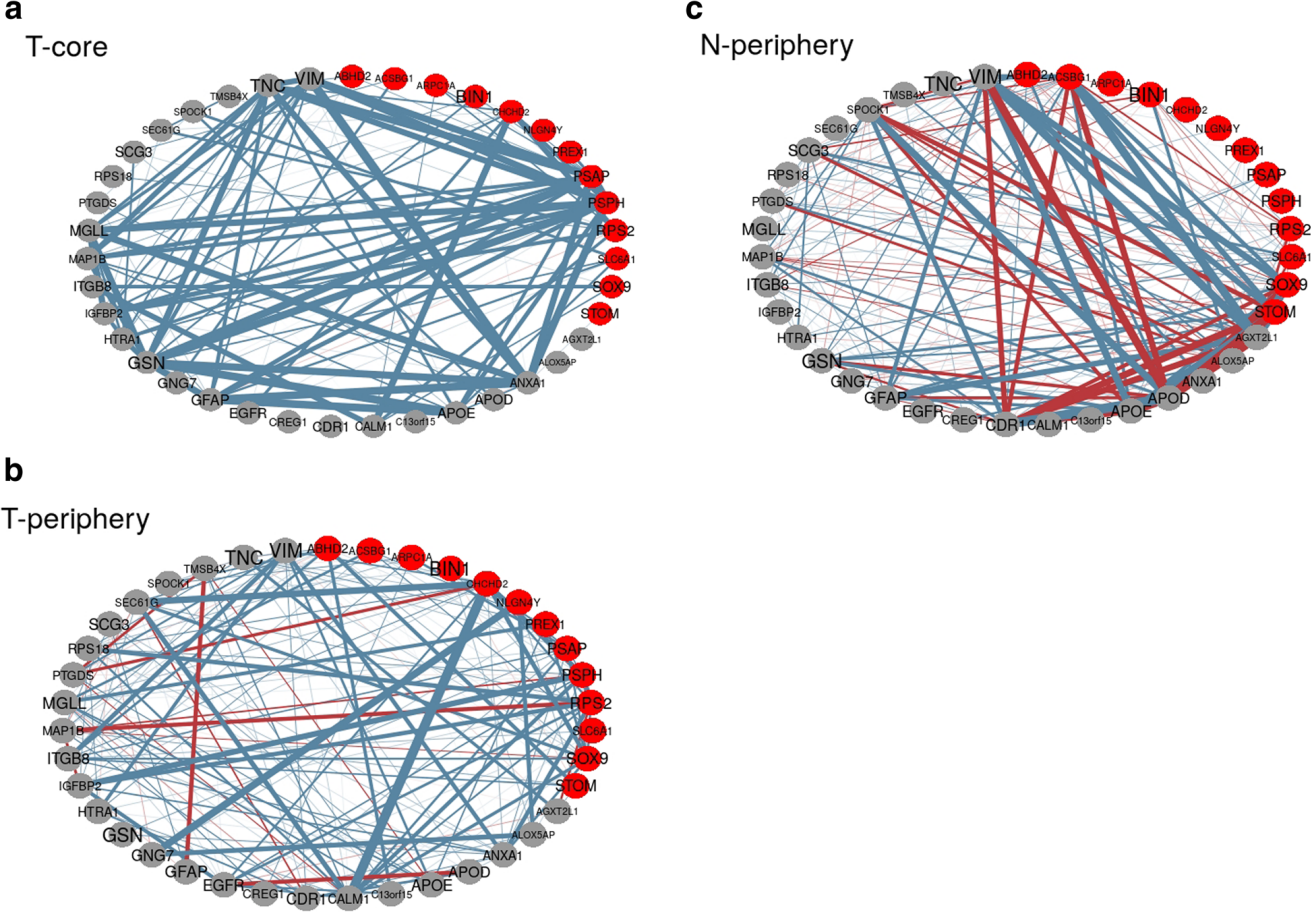
Correlation networks of the genes selected by Model III, in the three cell categories used (**a**, T-core, tumor core neoplastic astrocytes; **b**, T-periphery, tumor periphery neoplastic astrocytes; **c**, N-periphery, tumor periphery normal astrocytes) considering the variables selected by sparse logistic regression based on the twiner regularizer (gray coloured genes are genes selected in common by EN and twiner; red coloured genes are genes exclusively selected by twiner); blue lines represent positive correlations between genes, whereas red lines stand for negative correlations, with the thickness indicating the strength of the correlation

After gene selection, the correlation networks for the three astrocyte cell populations evaluated through twiner were obtained (Fig. 5), as a means to disclose the biological interrelationships within the gene signature extracted. For simplicity in graphical representation, only correlations above 0.2 are displayed. Blue lines represent positive correlations between genes, whereas red lines stand for negative correlations, with the thickness indicating the strength of the correlation. It can be noticed that despite the differences encountered for tumor core and periphery neoplastic astrocyte cell populations, the gene correlation network obtained for the tumor periphery normal cell population, as expected, is markedly different from the other two networks. The gene networks obtained, along with their similarities and contrasts, shall now be matter for further investigation regarding their role in GBM.

**Fig. 6:**
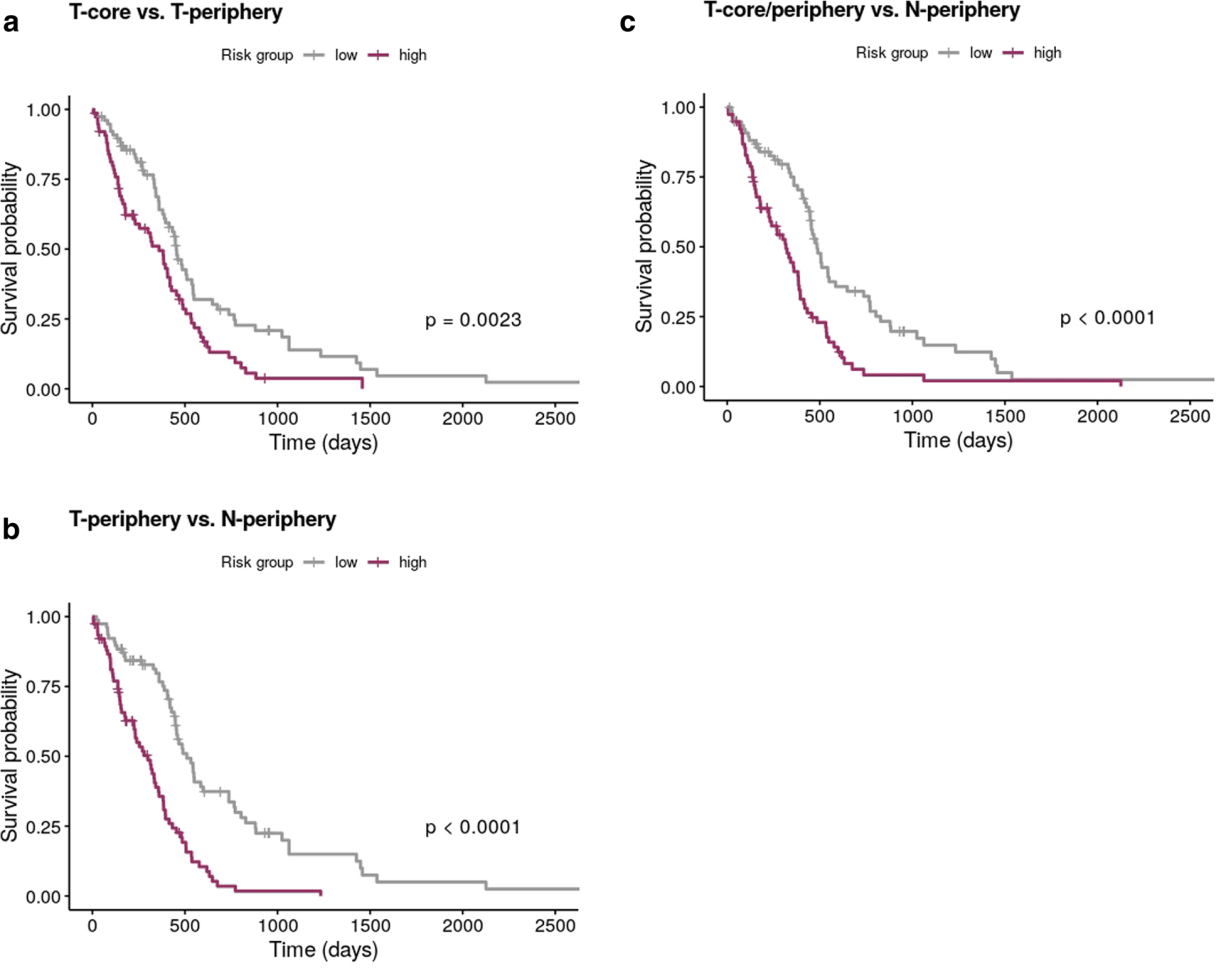
Kaplan-Meier survival curves obtained for bulk GBM RNA-seq data based on the variables selected by **a** Model I and **b** Model II via EN, and **c** Model III via twiner, showing significance given by the p-value for the three case scenarios in the separation between high (purple) and low (gray) risk patients (T-core, tumor core neoplastic astrocytes; T-periphery, tumor periphery neoplastic astrocytes; N-periphery, tumor periphery normal astrocytes)

The biological relevance of the genes signatures obtained through EN and twiner was verified on a survival dataset from a RNA-Seq bulk GBM population from the TCGA. For the three case studies evaluated, the survival curves obtained (Fig. 6) for Model I (T-core vs. T-periphery) and II (T-periphery vs. N-periphery) via EN, and Model III (T-core/periphery vs. N-periphery) via twiner show a statistically significant separation between high- and low-risk patients.

**Fig. 7:**
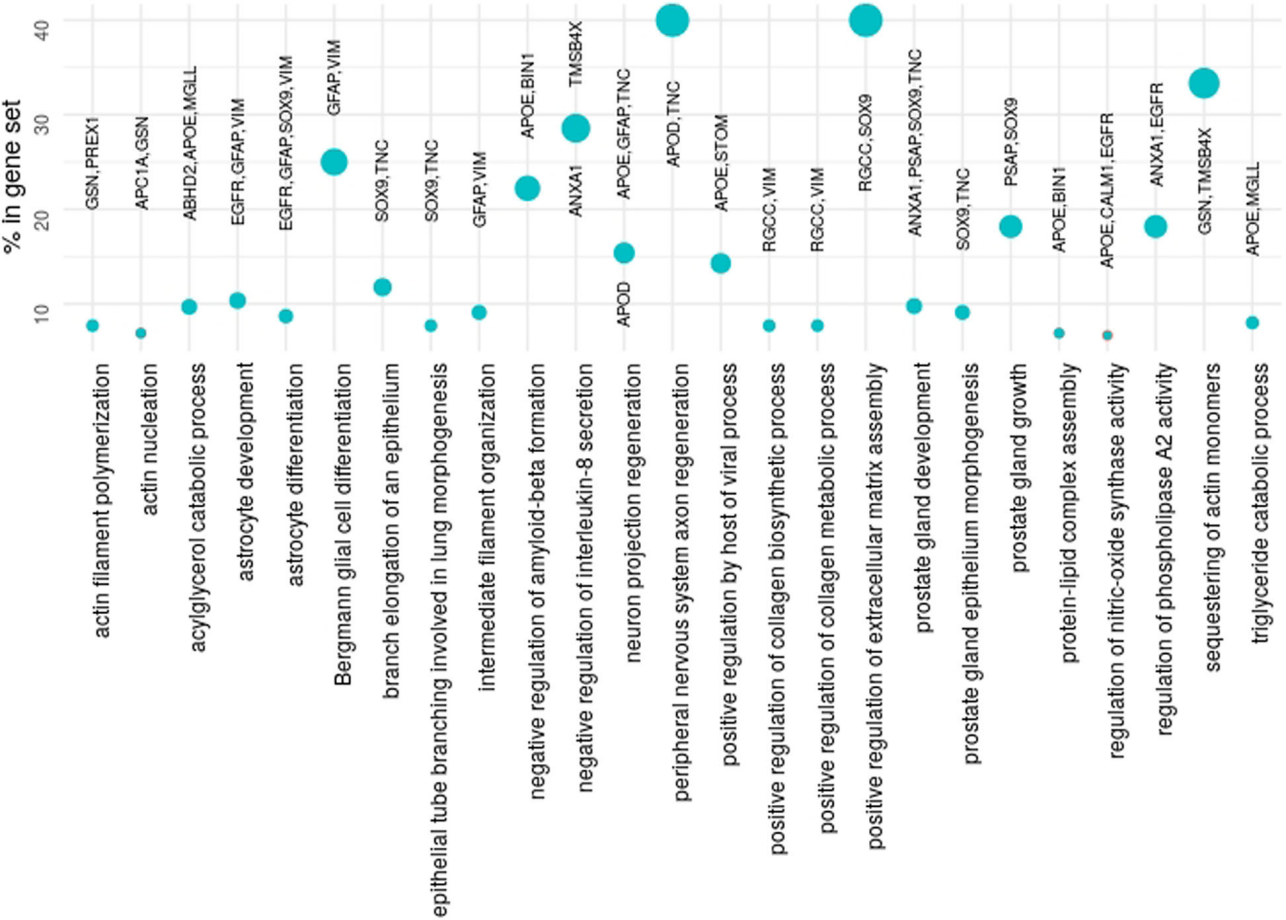
Percentage of the genes exclusively selected by twiner in the gene set associated to the GO terms found by GO analysis, sorted by increasing false discovery rate (from top to bottom)

A further GO enrichment analysis on the genes selected by Model III via twiner enabled the association of the genes present in the gene set with biological process GO terms (Fig. 7). From the list of 273 GO terms enriched, the top 25 given by the percentage of genes in the gene set associated to the term are listed, and sorted by increased false discovery rate (from top to bottom). From the genes selected, known markers in glioma and GBM, namely *SOX9* and *EGFR* [5, 19–21], are here associated to astrocyte development and differentiation.

Therefore, twiner enabled the selection of genes with a similar behaviour in the gene networks of neoplastic cells from tumor core and infiltrating neoplastic cells from the periphery through an accurate classification of GBM cells. Their relevance in GBM is supported by their significance in survival outcomes, and their association with relevant GO terms.

## Discussion

After model evaluation and gene selection, an attempt to biologically interpret the association between the gene signature obtained and GBM based on previous reports was made. Among the genes selected by Model I, discriminating between tumor core and periphery neoplastic astrocytes, 3 genes up-regulated in GBM infiltrating tumor cells with functions involving the invasion of the interstitial matrix were also pointed by Darmanis et al. (2017) [5], namely: *ATP1A2*, a NA ^+^/K ^+^ ATPase involved in size regulation; *PRODH*, related to proline catabolism and might contribute to increase ATP energy demands of migrating cells; and *FGFR3*, inducing increased infiltrating cell expression of cell survival signaling [5].

Among the genes always selected by Model II, discriminating between tumor periphery neoplastic astrocytes and normal tumor periphery astrocytes, the epidermal growth factor receptor (*EGFR*), up-regulated in neoplastic periphery astrocytes over normal periphery astrocytes, is a previously reported significantly mutated gene in GBM [20, 21].

Annexin 1 (*ANXA1*) is a member of the annexin superfamily of Ca2+ and phospholipid binding proteins, associated to the regulation of phospholipse A2 activity and negative regulation of interleukin-8 secretion in our GO analysis (Fig. 7), and up-regulated in neoplastic periphery (infiltrating) astrocytes (Table 2). *ANXA1* was shown to promote GBM tumor growth and progression and is more highly expressed in poorly differentiated human primary gliomas compared with lower grade tumors [22]. A hypomethylation signature consistently predicting poor prognosis in GBM was found to be closely associated with the transcriptional status of an EGFR/VEGFA/ANXA1-centered gene network [23]. *ANXA1* was also found to be correlated with *IGFBP2* (insulin-like growth factor-binding protein 2), a circulating biomarker for cancer diagnosis and a potential immunotherapeutic target, also belonging to the gene signature identified by Model II. *IGFBP2* was also found up-regulated in high-grade glioma and GBM and downregulated in *IDH* mutant glioma [24].

The serine protease *HTRA1*, down-regulated in neoplastic periphery (infiltrating) astrocytes in our analysis, is a binding partner of the macrophage migration inhibitory factor (MIF), both present in astrocytes, and whose functional binding modulates astrocytic activities in development and disease of the central nervous system (CNS) [25].

Regarding the genes selected by Model III via the EN and twiner regularizers, classifying cells into neoplastic (tumor core and periphery) and normal periphery astrocytes, not surprisingly many genes were selected in common with Model II (Table 2; Fig. 4), also classifying cells into neoplastic and normal astrocytes. By accounting for the periphery neoplastic astrocytes in the neoplastic class, Model III was intended to extract gene signatures shared by tumor core and periphery astrocytes. The novelty introduced by twiner regularization, on the other hand, aimed at extracting genes with a similar correlation pattern across the two neoplastic astrocyte populations (periphery and tumor core), that would not be selected otherwise. Beside improved model performance, this brings an obvious interpretability advantage in which concerns tumor heterogeneity over sparse classification via EN.

Therefore, particular attention will be given to the genes exclusively selected by Model III via the twiner regularizer, i.e., less penalized genes in the feature selection procedure, and expected to provide insight to therapy research on putative targets for multiple neoplastic clones. *CHCHD2* shows a particularly lower weight (Fig. 4), meaning that its correlation pattern across tumor core and periphery neoplastic astrocytes is more similar compared to the other genes, therefore being less penalized in sparse classification, and indeed being selected as relevant in the distinction between neoplastic (tumor core and periphery) and normal periphery astrocytes. Coamplification of *CHCHD2* and the well-known GBM marker *EGFR*, also included in the gene signature, has been reported in glioma [26, 27].

The transcriptomic factor *SOX9* was also exclusively selected by twiner. It is involved in brain development and lineage specification, and has a established oncogenic role in gliomas [5, 19].

*PSAP*, which together with *CHCHD2* presented the lowest weights (Fig. 4), has been pointed as a target for glioma treatment, by promoting glioma cell proliferation via the TLR4/NF- *κ*B signaling pathway [28]. *PREX1* and *ABHD2* have also shown to promote tumor invasion and progression in glioblastoma [29, 30], while the tumor suppressor *BIN1* was found to be regulated by *HNRNPA2B1*, a putative proto-oncogene in GBM [31].

Given the numerical results and the links established between the gene signatures extracted by our analysis and previously reported GBM molecular features, as shown above, we expect our findings to foster biological and clinical validation studies on the molecular and network features disclosed.

## Conclusions

This work was designed to tackle GBM tumor heterogeneity through the identification of gene signatures across multiple cell populations based on regularized classification of transcriptomic data. Our analysis was able to translate high-dimensional scRNA-Seq data into concise and interpretable gene networks of putative molecular drivers in GBM. The results obtained open the window to a in depth evaluation on their role in GBM evolutionary dynamics, and treatment resistance.

## Methods

### Glioblastoma scRNA-Seq data

The transcriptomic data on a cohort of four primary GBM patients (IDH1-negative, grade IV) used in this work were obtained from http://www.gbmseq.org/. The scRNA-Seq data correspond to 3,589 cells sequenced over 23,368 genes, from both tumor core and peritumoral brain tissues (Fig. 2), comprising neoplastic cells and representatives from each of the major CNS cell types (vascular, immune, neuronal, and glial). Cells were labeled regarding their tissue of origin (tumor core vs. peritumoral) and cellular type (neoplastic vs. non-neoplastic). Labels of cells were obtained by combining multiple analysis encompassing dimension reduction and clustering techniques, followed by inspection of de-regulated genes with a established role in GMBs and gliomas, and comparison with bulk RNA-Seq data. For validation of the cells’ location (tumor core or surrounding) hypoxic genes were investigated, which were found to be significantly more expressed within the tumor core cells.

### Sparse logistic regression

Binary sparse logistic regression was chosen as a classification strategy to extract gene signatures from GBM cell populations. Given a set of *p* independent variables (genes) {***X***_***i***_}_*i*=1,...,*n*_ for observation *i*, the expression has been corrected in the comment immediately above and a binary outcome vector **Y**={*Y*_*i*_}_*i*=1,...,*n*_, with classes ‘1’ and ‘0’ corresponding to different GBM clones, the parameters of the sparse logistic model are estimated by maximizing the log-likelihood function

1 $$ {\begin{aligned} l({\boldsymbol{\beta}}) = \sum_{i=1}^{n} \left\{y_{i} \log P (Y_{i}=1|\mathbf{X}_{i}) + (1 - y_{i}) \log \left[1 - P (Y_{i}=1|\mathbf{X}_{i})\right]\right\} + F({\boldsymbol{\beta}}), \end{aligned}}  $$

where ***β***=(*β*_1_,*β*_2_,…*β*_*p*_) are the regression coefficients associated with the *p* independent variables, and *P*(*Y*_*i*_=1|**X**_*i*_) is the probability of belonging to class 1 for observation *i*, given by

2 $$ P ({Y}_{i}=1|\mathbf{X}_{i})=\frac{\exp\left(\mathbf{X}_{i}^{T} {\boldsymbol{\beta}}\right)}{1+\exp\left(\mathbf{X}_{i}^{T} {\boldsymbol{\beta}}\right)}.  $$

For the elastic net (EN), the regularization term *F*(***β***) in Eq. 1 takes the form

3 $$ F(\boldsymbol{\beta}) = \lambda \Big\{\alpha \Vert \boldsymbol{\beta} \Vert_{1} + (1-\alpha) \Vert {\boldsymbol{\beta}} \Vert^{2}_{2} \Big\},  $$

with *α* controlling the balance between the *l*_1_ (LASSO) and *l*_2_ (Ridge) penalties, and the tuning parameter *λ* controlling the strength of the penalty.

Lopes et al. (2019) [17] proposed the *twin networks recovery* (twiner) penalty, a regularizer based on the EN penalty and the pairwise correlations between variables in two different datasets, with the specific goal of weighting the variables based on their similarities across two different diseases. The twiner regularization term in Eq. 1 becomes

4 $$  F(\boldsymbol{\beta}) = \lambda \left\{ \alpha \Vert \mathbf{w} \circ \boldsymbol{\beta} \Vert_{1} + (1-\alpha) \Vert \mathbf{w} \circ \boldsymbol{\beta} \Vert^{2}_{2} \right\},  $$

with **w**=(*w*_1_,...,*w*_*j*_,...,*w*_*p*_), *j*=1,...*p*, representing the weights that control the effect of *λ* in each coefficient *β*_*j*_, and ∘ representing the element wise (or Hadamard) product.

The construction of **w** for the twiner regularizer is based on the correlation matrices for classes *A* and *B*, $\Sigma _{A} = \left [{\boldmath {\sigma }}_{1}^{A},...,{\boldmath {\sigma }}_{p}^{A}\right ]$ and $\Sigma _{B} = \left [{\boldmath {\sigma }}_{1}^{B},...,{\boldmath {\sigma }}_{p}^{B}\right ]$, respectively, where each column ${\boldmath {\sigma }}_{j} \in \mathbb {R}^{p}$ represents the correlation of each gene *j*=1,…,*p* with the remaining genes in the dataset. The weight for gene *j*, *w*_*j*_, to be used in the twiner regularizer (Eq. 4), is given by the angle of the resulting correlation vectors $\boldmath {\sigma }_{j}^{A}$ and $\boldmath {\sigma }_{j}^{B}$, normalized by the maximum value in **w**. The lower the weight for gene *j*, the lower the penalty associated to that gene.

In the example of application provided in [17], a smaller penalty was imposed for those genes with a similar correlation pattern with the remaining ones across independent breast and prostate RNA-Seq data matrices. The relevance of these genes in the classification outcome was assessed by sparse logistic regression based on the EN penalty, where classes are tumor (breast and prostate) and normal (breast and prostate) tissue samples. The final goal is to assess whether genes exhibiting a similar behavior in the two genes networks are putative biomarkers for the two diseases.

#### Classification of GBM scRNA-Seq data

**Fig. 8:**
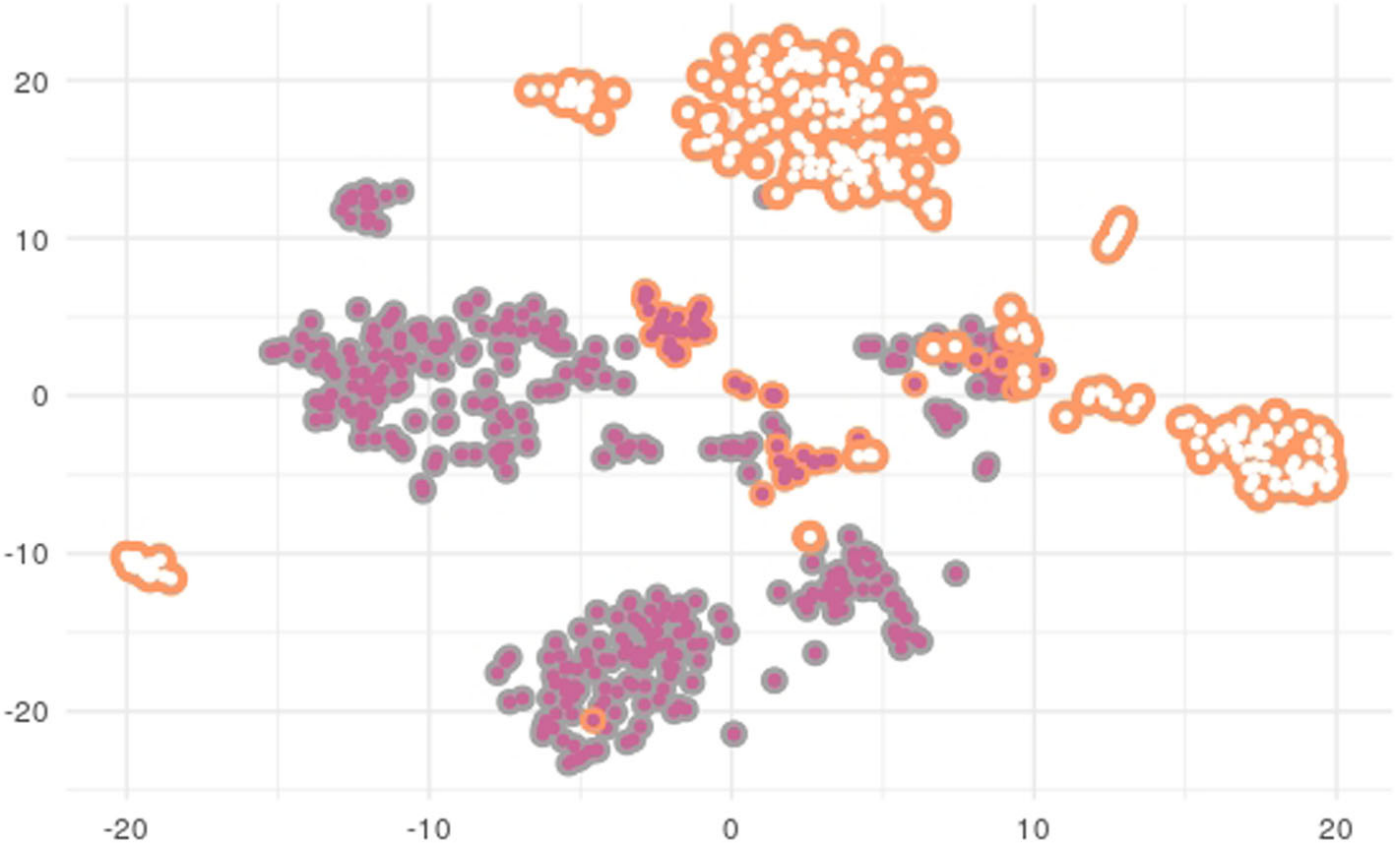
2D-tSNE representation of all cells ( tumor core neoplastic astrocytes;  tumor periphery neoplastic astrocytes;  tumor periphery normal astrocytes), demonstrating separation by cell type (neoplastic and normal) and location (tumor core and periphery)

Sparse logistic regression models using the EN and twiner regularizers were built based on GBM scRNA-Seq to identify gene signatures across GBM cell populations. The cells chosen for modeling were neoplastic and normal astrocytes from the tumor periphery and neoplastic astrocytes from the tumor core (Fig. 2), given their representativeness across tumor locations. A 2D t-distributed stochastic neighbor embedding (tSNE) representation of cells can be found in Fig. 8, where it is clear that infiltrating neoplastic astrocytes from the tumor periphery stand closer to the data cloud formed by tumor core neoplastic astrocytes.

Three classification strategies were defined to model the above cell populations with distinct goals. A schematic representation of the classification models generated van be found in Fig. 1. Model I takes as class 1 the neoplastic astrocytes from the tumor core (T-core), and as class 0 the neoplastic astrocytes from the periphery (T-periphery), with the goal of identifying genes that discriminate between the two classes, e.g., those making tumor cells capable to migrate beyond the tumor environment. Model II looks only at tumor periphery cells, by considering as class 1 the neoplastic astrocytes (T-periphery) and as class 0 the normal (non-neoplastic) ones (N-periphery), aiming at disclosing cancer drivers that make astrocytes from the periphery distinguishable in neoplastic and normal cells. Finally, Model III takes as class 1 the neoplastic astrocytes irrespective of their tissue of origin (T-core and T-periphery, i.e., both from the tumor core and periphery), and as class 0 the normal (non-neoplastic) astrocytes (N-periphery), with the goal of extracting the relevant genes in the classification of cells into neoplastic and normal astrocytes.

Sparsity and gene selection were enforced by the EN regularizer in the three models. Additionally, the twiner regularizer was applied in Model III to extract the variables that are similarly correlated in the genes network in neoplastic astrocytes from both the periphery and tumor core, and that are found to play a role in the discrimination between neoplastic (tumor and periphery; class 1) and normal astrocytes (class 0), the later only represented in the tumor periphery. With this strategy we expect to unveil shared molecular signatures between neoplastic astrocytes irrespective of their tissue of origin.

For the three classification modeling strategies, the optimization of the model parameters *λ* and *α* (Eq. 4) based on the mean squared error (MSE) was performed by 10-fold cross-validation (CV) on the full dataset. Varying *α* values (1 > *α* > 0) were tested, with the one yielding the lowest MSE being selected for further analysis. Models I, II and III were generated 1000 times based on data partitions accounting for three quarters of randomly selected cell samples for model training and the remaining samples for testing, while ensuring representativeness of both classes in the two sets. The performance of the models was assessed by the median MSE, area under the Precision-Recall curve (AUC), and the number of misclassifications in the training and test sets. The identity of the genes selected in more that 75% of the runs was kept for further biological interpretation in the context of GBM.

Besides sparse logistic regression through the EN penalty, a Naïve Bayes (NB) classifier was used as a benchmark method in Model III against sparse logistic regression via EN and twiner. NB classifiers assume conditionally independence of the features given the class, which simplifies enormously the estimation of the probability density functions. This technique is thus especially appropriate for high-dimensional problems and therefore suitable to this type of data. Although NB assumptions are not usually met, NB continues to outperform more sophisticated classifiers, which makes it a good benchmark candidate for comparison purposes.

To compare the different models, the NB classifier was applied (using a Gaussian approximation for the probability density functions of each feature) to the subsets of variables selected by EN, and twiner.

In order to further biologically validate the genes selected as relevant in the disease, a survival analysis was performed using the Cox regression model [32] based on the genes selected in Model III by both EN and twiner. The goal was to assess whether the genes selected are significant in the discrimination of high- and low-risk groups of patients, defined by the median of the fitted relative risk, based on the Log-Rank test via the Kaplan-Meier estimator [33]. This analysis was performed based on 139 bulk GBM RNA-Seq samples available from The Cancer Genome Atlas (TCGA) data portal (https://cancergenome.nih.gov/).

Finally, a Gene Ontology (GO) hypergeometric enrichment analysis [34] was performed to identify from the genes selected those associated to GO biological process terms.

Sparse logistic modeling and survival analysis was performed using the glmnet R package [35] implemented in the free R statistical software [36]. The *w* vector built for the twiner regularizer was introduced as penalty factor in the glmnet function. The limma Bioconductor R package [37] was used to identify differentially expressed genes across the tumor tissues. The association between the genes selected and GO biological terms was obtained using the functional enrichment analysis provided by STRING [34].

## Data Availability

All the implementations described can be found in a R Markdown document available at http://web.tecnico.ulisboa.pt/susanavinga/GBM/, which allows full reproducibility and adaptation to new datasets.

## Abbreviations

AUC: Area under the curve
GBM: Glioblastoma
CNS: Central nervous system
CV: Cross-validation
EN: Elastic net
GO: Gene Ontology
LASSO: Least absolute shrinkage and selection operator
MSE: Mean squared error
NB: Naï
ve Bayes; RNA-Seq: RNA sequencing
scRNA-Seq: single-cell RNA sequencing
TCGA: The Cancer Genome Atlas
tSNE: t-distributed stochastic neighbor embedding
twiner: Twin networks recovery
